# Identification of a novel microRNA signature associated with intrahepatic cholangiocarcinoma (ICC) patient prognosis

**DOI:** 10.1186/s12885-015-1067-6

**Published:** 2015-02-18

**Authors:** Mei-Yin Zhang, Shu-Hong Li, Guo-Liang Huang, Guo-He Lin, Ze-Yu Shuang, Xiang-Ming Lao, Li Xu, Xiao-Jun Lin, Hui-Yun Wang, Sheng-Ping Li

**Affiliations:** 1 State Key Laboratory of Oncology in South China, Sun Yat-Sen University Cancer Center, Guangzhou, 510060 China; 2 National Collaborative Innovation Center for Cancer Medicine, Sun Yat-Sen University Cancer Center, Guangzhou, 510060 China; 3 Department of Hepatobiliary Oncology, Sun Yat-Sen University Cancer Center, Guangzhou, 510060 China; 4 Sino-American Cancer Research Institute, Guangdong Medical College, Dongguan, 523808 China

**Keywords:** microRNA, Intrahepatic cholangiocarcinoma, Biomarker, Prognosis

## Abstract

**Background:**

The clinical significance of microRNAs (miRNAs) in intrahepatic cholangiocarcinoma (ICC) is unclear. The objective of this study is to examine the miRNA expression profiles and identify a miRNA signature for the prognosis of ICC.

**Methods:**

Using a custom microarray containing 1,094 probes, the miRNA expression profiles of 63 human ICCs and nine normal intrahepatic bile ducts (NIBD) were assessed. The miRNA signatures were established and their clinical significances in ICC were analyzed. The expression levels of some miRNAs were verified by quantitative real-time RT-PCR (qRT-PCR).

**Results:**

Expression profile analysis showed 158 differentially expressed miRNAs between ICC and NIBD, with 77 up-regulated and 81 down-regulated miRNAs. From the 158 differentially expressed miRNAs, a 30-miRNA signature consisting of 10 up-regulated and 20 down-regulated miRNAs in ICC was established for distinguishing ICC from NIBD with 100% accuracy. A separate 3-miRNA signature was identified for predicting prognosis in ICC. Based on the 3-miRNA signature, a formula was constructed to compute a risk score for each patient. The patients with high-risk had significantly lower overall survival and disease-free survival than those with low-risk. The expression level of these three miRNAs detected by microarray was verified by qRT-PCR. Multivariate analysis indicated that the 3-miRNA signature was an independent prognostic predictor.

**Conclusions:**

In this study, a 30-miRNA signature for distinguishing ICC from NIBD, and a 3-miRNA signature for evaluating prognosis of ICC were established, which might be able to serve as biomarkers for prognosis of ICC. Further studies focusing on these miRNAs may shed light on the mechanisms associated with ICC pathogenesis and progression.

## Background

Intrahepatic cholangiocarcinoma (ICC) is a high-grade malignant neoplasm originating from the small bile duct epithelium in the liver [1], and is the second most common intrahepatic primary tumor after hepatocellular carcinoma (HCC). It comprises 5.4% of primary liver neoplasms [2] and its incidence is increasing [3,4]. Curative resection is still considered to be the only effective treatment; however, the 5-year survival rate of patients with ICC after surgery is low, at only 25% to 35% in most studies [5] and the recurrence rate at 5 years is as high as 67.9% [6]. Furthermore, there is no molecular marker for predicting the prognosis of patients with ICC in clinical practice and studies on molecular makers in ICC patients are limited. Therefore, identifying molecular marker for prognosis of ICC patients is an urgent need in clinical practice.

MicroRNAs (miRNAs) are small (18–25 nucleotides) non-coding single-stranded RNA molecules that negatively regulate gene expression by base-pair matching with the 3′ UTRs of target mRNAs [7] and are reported to be involved in a variety of physiological and pathological processes, including development, differentiation, apoptosis, proliferation and carcinogenesis [7,8]. Previous studies have shown that miRNAs are dysregulated in many cancers and the aberrantly expressed miRNAs might serve as diagnostic and prognostic biomarkers for various tumors [9-17]. To date, there have only been three studies on miRNA expression profiles in ICC tissue samples: the first identified a 38-miRNA signature in 27 ICC tissues for distinguishing ICC from normal tissue [18], the second established a 23-miRNA signature associated with tumor subtypes and prognosis in 23 ICCs and combined hepatocellular-cholangiocarcinomas [19], and the third found that different miRNA profiles correlated with the histological grade and the subtype of 15 ICCs induced by liver fluke *Opisthorchis viverrini* [20]. Although miRNA profile studies on ICC tissues are very limited, there are a number of single miRNA expression studies on ICC tissues and cell lines. For examples, some miRNAs were identified to be involved in ICC cell growth and apoptosis (miR-31) [21], migration or invasion (miR-376c and miR-214) [22,23], metastasis [24], and epithelial to mesenchymal transition (EMT) (miR-200c and miR-204) [19,25]. However, the clinical significance of miRNA signatures in ICC still needs to be elucidated because of small sample sizes and very limited studies. In this study, we analyzed the miRNA expression profiles in 63 patients with ICC and nine normal intrahepatic bile ducts (NIBD) using a custom microarray containing probes for 1,094 miRNAs. The aim of the present study was to identify miRNA signatures that could be used as a biomarker for prognosis in patients with ICC and provided insight for further investigation into the mechanisms involved in ICC development and progression.

## Methods

### Patients

All 63 patients (44 men, 19 women) with ICC who underwent resection in the Hepatobiliary Department, Sun Yat-Sen University Cancer Center, between 1999 and 2010, were included in this study. The ICC was pathologically diagnosed at surgery and confirmed by a separate experienced pathologist in this study. None of these patients had received anticancer therapy, such as radiotherapy or chemotherapy, before surgery. After hepatectomy, the patients were not given any other therapies except the regular liver protection treatment. If patients had hepatitis B virus (HBV) infection, serum alanine aminotransferase (ALT) elevation (>40 U/L) and serum positive for hepatitis B surface antigen (HBsAg), hepatitis B extracellular antigen (HBeAg) and HBV DNA, they would undergo antiviral therapy. The NIBD were collected as normal control from nine patients with HCC who underwent hepatectomy at Sun Yat-Sen University Cancer Center between June and July in 2011 and were confirmed histologically to be free of tumors. This study was reviewed and approved by the Human Research Ethics Committee at Sun Yat-Sen University Cancer Center, and written informed consent was obtained from patients.

The clinicopathologic information was obtained from chart review and is listed in Table 1. The histological grade (I-III) of tumor was determined according to the grading system proposed by Edmondson and Steiner. All of the patients were staged according to the American Joint Committee on Cancer Staging Manual (Seventh Edition).

**Table 1:** **Comparison of characteristics of patients with ICC in high- or low risk groups**

Characteristics		n (%)	No. of patients (%)		*P*value
			High-risk group	Low-risk group	
Gender	Male	44 (69.8)	19 (65.5)	25 (73.5)	0.490
	Female	19 (30.2)	10 (34.5)	9 (26.5)	
Age (years)	<50	23 (36.5)	12 (41.4)	11 (32.4)	0.458
	≥50	40 (63.5)	17 (58.6)	23 (67.6)	
ALT (U/L)	≤40	48 (76.2)	24 (82.8)	24 (70.6)	0.258
	>40	15 (23.8)	5 (17.2)	10 (29.4)	
AST (U/L)	≤45	55 (87.3)	26 (89.7)	29 (85.3)	0.716
	>45	8 (12.7)	3 (10.3)	5 (14.7)	
TBIL (mmol/L)	≤20.5	54 (85.7)	27 (93.1)	27 (79.4)	0.160
	>20.5	9 (14.3)	2 (6.9)	7 (20.6)	
HBsAg	Negative	35 (55.6)	17 (58.6)	18 (52.9)	0.651
	Positive	28 (44.4)	12 (41.4)	16 (47.1)	
AFP (ng/mL)	≤25	59 (93.7)	26 (89.7)	33 (97.1)	0.326
	>25	4 (6.3)	3 (10.3)	1 (2.9)	
CA199 (U/L)	≤35	27 (42.9)	11 (37.9)	16 (47.1)	0.466
	>35	36 (57.1)	18 (62.1)	18 (52.9)	
CEA (ng/mL)	≤5	46 (73.0)	19 (65.5)	27 (79.4)	0.216
	>5	17 (27.0)	10 (34.5)	7 (20.6)	
Cirrhosis	Yes	20 (31.7)	10 (34.5)	10 (29.4)	0.666
	No	43 (68.3)	19 (65.5)	24 (70.6)	
Histological grade	I + II	26 (41.3)	12 (41.4)	14 (41.2)	0.987
	III	37 (58.7)	17 (58.6)	20 (58.8)	
T stage	T1	35 (55.6)	16 (55.2)	19 (55.9)	0.630
	T2a	5 (7.9)	1 (3.4)	4 (11.8)	
	T2b	17 (27.0)	9 (31.0)	8 (23.5)	
	T3	6 (9.5)	3 (10.3)	3 (8.8)	
N stage	N0	44 (69.8)	17 (58.6)	27 (79.4)	0.073
	N1	19 (30.2)	12 (41.4)	7 (20.6)	
TNM stage	I + II	38 (60.3)	14 (48.3)	24 (70.6)	0.071
	III + IV	25 (39.7)	15 (51.7)	10 (29.4)	

ALT, alanine aminotransferase; AST, aspartate aminotransferase; TBIL, total bilirubin; HBsAg, hepatitis B surface antigen; AFP, alpha-fetoprotein; CA19-9, carbohydrate antigen 19–9; CEA, carcinoembryonic antigen.

### Follow-up

The patients were followed monthly in the first 2–3 months after surgery, then every 2–3 months in the first year and 3–6 months thereafter. When tumor recurrence or metastasis was suspected, further examinations including magnetic resonance imaging (MRI), positron emission tomography/computed tomography (PET/CT) and biopsies were performed. Besides the clinic interview, specialized staff followed patients via telephone. The follow-up data of each patient was regularly updated. The median follow-up time of the 63 patients was 18.3 months (ranging from 1 to 67.9 months). The overall survival (OS) was computed from the date of hepatectomy to the date of death, and disease-free survival (DFS) was computed from the date of hepatectomy to the first relapse, distant metastasis, or death. During this follow-up period, all the deaths were cancer-related.

### Generation of custom miRNA microarray

We conducted the probe design with the protocol as described by Wang and colleagues [26]. All of 1,112 human mature miRNAs (release 16) in the miRBase database were used for designing probes, but only 1,094 human miRNA probes were successfully designed because of the high homology between some miRNAs. The miRNA microarray was made in-house according to the protocols previously reported by us [15].

### RNA extraction and microarray experiments

The paraffin-embedded tissues from the 63 patients with ICC were obtained from the Department of Pathology, Sun Yat-Sen University Cancer Center. We cut five sections with 10 μm thickness from each patient, and mounted them onto glass slides. Tumor areas (containing > 90% tumor tissue) were scraped off with a scalpel under a microscope and collected in nuclease-free microcentrifuge tubes. The NIBDs were peeled off from the resected liver. Total RNA was extracted from ICC and NIBD with an acid phenol-chloroform extraction method, followed by ethanol precipitation, as described previously [27]. Quantity and quality of RNA were measured by using a NanoDrop™ 1000 (Thermo Fisher Scientific, MA, USA) spectrophotometer.

Total RNA (2.5 μg) from each sample was used for labeling with pCp-DY647 (Dharmacon, Lafayette, CO, USA) and hybridized in accordance with published protocols [15]. After hybridization, the microarray was scanned with a LuxScan 10 K Microarray Scanner (CapitalBio, Beijing, China) and the scanned images were gridded by using GenePix Pro 6.0 software (Axon Instruments, Foster City, CA, USA).

### Quantitative reverse transcription PCR (qRT-PCR)

The reverse transcription (RT) was carried out in a volume of 12.5 μL containing 500 ng of total RNA, 5 nmol/L of Bulge-Loop™ miRNA RT specific primers (RiboBio Co., Guangzhou, PR China), 0.2 mmol/L dNTP, 40 U RNase inhibitor and 20 U M-MLV reverse transcriptase (Promega, Madison, WI, USA) at 42°C for 60 minutes. The quantitative PCR (qPCR) reaction was performed in 15 μL volume with 3 μL of RT products, 500 nmol/L each of Bulge-Loop miRNA forward specific primer and universal reverse primer, and 6.75 μL of GoTaq qPCR Master Mix Reagent (Promega) on LightCycler480 instrument (Roche Diagnostics, Penzberg, Germany). U6 snRNA was used as the internal control. The PCR amplification was performed according to the manufacturer’s instruction. The comparative Ct method (ΔΔCt) was used to quantify miRNA expression, and the relative quantification was calculated as 2^-ΔΔCt^ to represent expression changes of miRNA between ICC and NIBD.

### Data process and statistical analysis

The raw microarray data were normalized by using Quantile Normalization Software and then processed by log 2 transformation. The normalized microarray data are available at National Center for Biotechnology Information Gene Expression Omnibus (accession number GSE53870). The differential expression of miRNA was analyzed by Significance Analysis of Microarrays (SAM, Stanford University, CA) and Student’s t test. Hierarchical clustering analysis (HCL) was performed to assess differential expressions of miRNAs between ICC and NIBD and miRNAs of interest by using Multi Experiment Viewer (MEV, version 4.2).

Univariate Cox regression analysis was applied to search for miRNAs associated with overall survival (OS). Multivariate Cox regression analysis was carried out to establish a signature and develop a formula for OS prediction with miRNAs that had *P* < 0.05 in univariate analysis. The formula was used to calculate the risk score for each patient. The risk score = sum of coefficient of each miRNA × expression level of corresponding miRNA in the signature. The patients were thus divided into a high-risk group and a low-risk group by using the median risk score as the threshold value. Kaplan-Meier analysis and the log-rank test were employed to assess OS and disease-free survival (DFS) of the two groups. The chi-square test or Fisher’s exact test were used to analyze the correlations between clinical characteristics and miRNA signature. Finally, multivariate Cox regression analysis was used to access if the miRNA signature was an independent prognostic factor for OS. The SPSS 16.0 (Inc., Chicago IL, USA) and GraphPad Prism 5 (San Diego, CA, USA) programs were used for statistical analysis and data plotting.

## Results

### Identification of a 30-miRNA signature to discriminate ICC and NIBD

**Figure 1:**
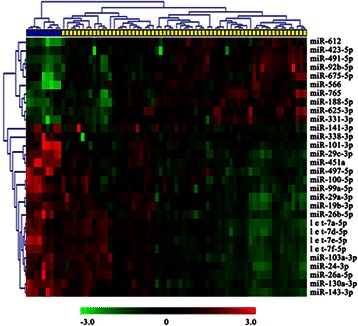
**Hierarchical clustering analysis of ICC and NIBD samples with 30-miRNA signature.** The 30-miRNA signature was identified from 158 differentially expressed miRNAs between 63 ICCs and nine NIBDs. Heat map representing the expression level of each probe (rows) in the 30-miRNA signature (green color = low, and red color = high) in each sample (columns). The 63 ICCs and nine NIBDs were clustered into two groups by the 30-miRNA signature with 100% accuracy.

miRNA expression profiles of 63 ICCs and nine NIBD were detected using our custom miRNA microarray. SAM analysis (false discovery rate (FDR) was set to 0) revealed that there were 158 miRNAs with differential expression between ICC and NIBD samples. A total of 77 miRNAs were up-regulated and 81 were down-regulated in ICC tissues, relative to NIBD samples. To identify a signature to distinguish ICC from NIBD, miRNA with more than a 2-fold change and low *P* values were selected from the 158 miRNAs by using the SAM program (FDR = 0) and t test. A 30-miRNA signature was developed by class prediction and clustering, which reached the maximum correct classification rate (100%) for ICC and NIBD tissues (Figure 1). Of the 30 miRNAs (Table 2), 10 were up-regulated and 20 down-regulated in ICC. This result suggested that the 30 miRNA for distinguishing ICC from NIBD might be involved in ICC development and progression.

**Table 2:** **Summary of 30 miRNAs associated with distinguishing ICC from NIBD**

No.	miRNA	Mean Int. in ICC	Mean Int. in NIBD	Ratio (ICC/NIBD)	Expression in ICC
1	miR-566	8177	1264	6.47	Up
2	miR-423-5p	7091	1906	3.72	Up
3	miR-612	4114	1129	3.64	Up
4	miR-765	7351	2074	3.54	Up
5	miR-625-3p	5218	1504	3.47	Up
6	miR-491-5p	2957	917	3.23	Up
7	miR-188-5p	5692	1819	3.13	Up
8	miR-92b-5p	5022	1686	2.98	Up
9	miR-675-5p	20464	8093	2.53	Up
10	miR-331-3p	2537	1045	2.43	Up
11	miR-141-3p	1162	2405	0.48	Down
12	miR-497-5p	897	1961	0.46	Down
13	miR-29a-3p	4118	9536	0.43	Down
14	let-7a-5p	3491	8203	0.43	Down
15	miR-19b-3p	1647	3883	0.42	Down
16	miR-103a-3p	1490	3532	0.42	Down
17	miR-130a-3p	981	2398	0.41	Down
18	let-7d-5p	1616	3997	0.40	Down
19	miR-100-5p	1343	3682	0.36	Down
20	miR-26b-5p	924	2558	0.36	Down
21	let-7e-5p	988	2819	0.35	Down
22	miR-24-3p	2737	7954	0.34	Down
23	miR-101-3p	580	1685	0.34	Down
24	let-7f-5p	1414	4140	0.34	Down
25	miR-99a-5p	1093	3314	0.33	Down
26	miR-338-3p	543	2095	0.26	Down
27	miR-29c-3p	1379	5357	0.26	Down
28	miR-26a-5p	3213	14510	0.22	Down
29	miR-451a	941	5163	0.18	Down
30	miR-143-3p	2842	23456	0.12	Down

Int., Intensity.

### Identification of novel 3-miRNA signature associated with survival in ICC

**Figure 2:**
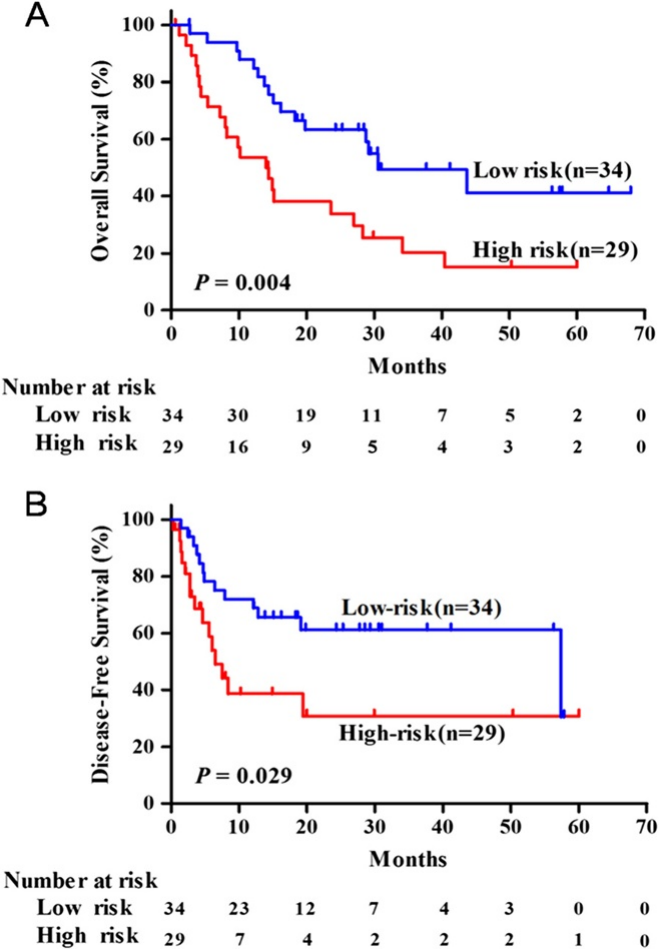
**Survival analysis of ICC patients in high- or low-risk groups.** According to the risk score of the 3-miRNA signature, ICC patients were divided into high- and low-risk groups. **(A)** Kaplan-Meier curve analysis of overall survival (OS) of ICC patients in high- and low-risk groups. **(B)** Kaplan-Meier curve analysis of disease-free survival (DFS) of ICC patients in high- and low-risk groups.

To identify miRNAs whose expression pattern is significantly associated with the prognosis of ICC, the miRNAs with expression in more than 10% of samples and displayed more than a 1.5-fold change in expression were screened by univariate Cox regression analysis. Three miRNAs (miR-675-5p, miR-652-3p and miR-338-3p) were found to be significantly associated with OS (*P* < 0.05). Of the three miRNAs, miR-675-5p was up-regulated and negatively associated with OS (hazard ratio [HR]: 2.562, confidence interval [CI]: 1.295-4.929), while the other two (miR-652-3p and miR-338-3p) were down-regulated and positively associated with OS (HR: 0.477, CI: 0.247-0.922; HR: 0.498, CI: 0.257-0.966, respectively). To find the best predictor for survival, we performed receiver operating characteristic (ROC) analysis on single miRNAs, as well as different combinations of the three miRNAs. In decreasing order of performance, the results showed that the predictive performance of the 3-miRNA signature is the best (area under the curve (AUC): 0.747, *P* = 0.002), followed by single miR-675-5p (AUC: 0.686, *P* = 0.021), the combination of miR-675-5p and miR-652-3p (AUC: 0.686, *P* = 0.021), the combination of miR-675-5p and miR-338-3p (AUC: 0.686, *P* = 0.021), single miR-652-3p (AUC: 0.622, *p* = 0.130), single miR-338-3p (AUC: 0.622, *P* = 0.130), and the combination of miR-652-3p and miR-338-3p (AUC: 0.587, *P* = 0.281). Next, a previously developed strategy [15] was used to establish a formula to calculate the risk score for every patient based on the expression level of the three miRNAs, weighted by regression coefficient: Risk Score = (0.93 × expression level of miR-675-5p) + (−0.726 × expression level of miR-652-3p) + (−0.688 × expression level of miR-338-3p). According to the risk score, patients were divided into a high-risk group and a low-risk group by the median signature risk score as the cut-off point. Since five patients with the same median risk score were designated into the low-risk group, there were 34 patients in the low-risk group and 29 in the high-risk group. Survival analysis showed that the patients in the low-risk group had 1- and 2-year survival rates of 88.1% and 57.4%, respectively, while the patients in the high-risk group had 1- and 2-year survival rates of 54.4% and 41.4%, respectively. The median OS was 14 months for the high-risk group compared with 26.5 months for the low-risk group (*P* = 0.004; Figure 2A). In addition, the median DFS was 4.4 months for the high-risk group and 17.3 months for the low-risk group (*P* =0.029; Figure 2B). Kaplan-Meier survival analysis of the patients in the two subgroups revealed that OS and DFS rates in the high-risk group were significantly lower than those in the low-risk group (Figure 2).

### Expression levels of 3-miRNA signature validated by RT-PCR

**Figure 3:**
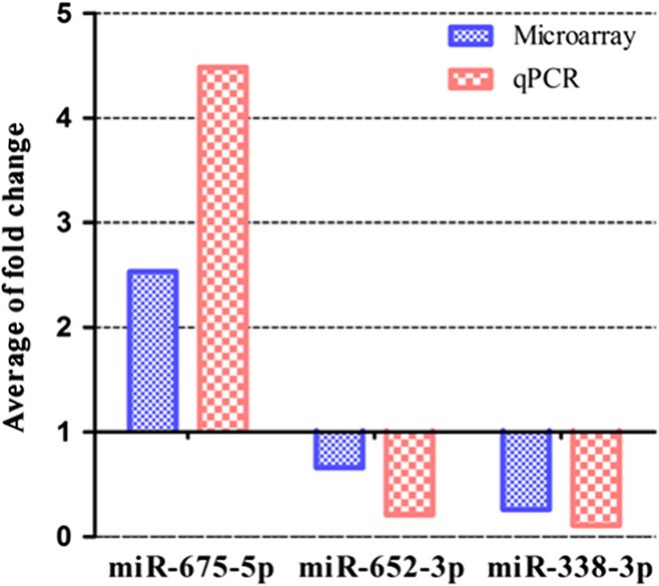
**The expression levels of three miRNAs detected with microarray were verified by qRT-PCR.** Histogram plot indicating that the expression levels of three miRNAs (miR-675-5p, miR-652-3p and miR-338-3p) measured by microarray were concordant with those by qRT-PCR, and Spearman correlation analysis showed the high correlations (see the Results section for details) between the expression levels of each miRNA detected by microarray and qRT-PCR.

To confirm the miRNA expression level detected by the microarray, we carried out qRT-PCR for miR-675-5p, miR-652-3p, and miR-338-3p in ICC samples and NIBD tissues. The results showed that the expression levels of the three miRNAs detected by microarray significantly correlated with those measured by qRT-PCR (miR-675-5p, R = 0.566, *P* = 0.0012; miR-652-3p, R = 0.761, *P* < 0.0001; miR-338-3p, R = 0.623, *P* = 0.0009) (Figure 3). These results show that miRNA levels detected by microarray are reliable and can be used for the further study.

### The relationship between 3-miRNA signature and clinicopathological features

We next explored whether the 3-miRNA signature was correlated with clinicopathological features of ICC (Table 1). With the chi-square test, the 3-miRNA signature was found to be marginally significantly with tumor-node-metastasis (TNM) stage (*P* = 0.071), while no statistically significant associations were observed between 3-miRNA signature and other clinicopathological features (Table 1).

### Univariate and multivariate Cox regression analysis of the 3-miRNA signature and clinical variables

To further verify whether the signature was an independent prognostic factor, the signature and clinical variables in all of the 63 patients with ICC were analyzed by Cox regression model. The univariate Cox regression analysis indicated that the 3-miRNA signature, the alpha-fetoprotein (AFP), T stage, N stage and TNM stage were significant predictors for OS (*P* = 0.006, *P* = 0.047, *P* = 0.007, *P* = 0.001 and *P* < 0.001, respectively). In the multivariate analysis, the 3-miRNA signature (HR: 2.13, 95% CI: 1.108 - 4.107; *P* = 0.023) and TNM stage (HR: 3.37, 95% CI: 1.733 - 6.651; *P* < 0.001) were independent prognostic factors for OS (Table 3).

**Table 3:** **Univariate and multivariate analysis of clinical features associated with overall survival**

Characteristics	HR (95.0%CI)	*P*value
UNIVARIATE ANALYSIS		
3-miRNA signature (high-risk vs. low-risk)	2.49 (1.300-4.750)	0.006
Gender (M vs. F)	1.49 (0.699-3.153)	0.303
Age (≥50 vs. <50)	1.30 (0.665-2.557)	0.439
ALT (>40 vs. ≤40)	0.69 (0.302-1.572)	0.375
AST (>45 vs. ≤45)	0.63 (0.220-1.781)	0.38
TBIL (>20.5 vs. ≤20.5)	0.57 (0.222-1.469)	0.245
HBsAg (Positive vs. Negative)	0.90 (0.475-1.714)	0.755
AFP (≤25 vs. >25)	3.43 (1.014-11.574)	0.047
CA19-9 (>35 vs. ≤35)	1.91 (0.977-3.746)	0.058
CEA (>5 vs. ≤5)	1.81 (0.909-3.611)	0.091
Cirrhosis (Yes vs. No)	1.32 (0.669-2.613)	0.442
Edmondson Steiner grade (I + II vs. III)	1.02 (0.533-1.953)	0.951
T stage (T2b + T3 vs. T1 + T2a)	2.48 (1.282-4.780)	0.007
N stage (N1 vs. N0)	3.07 (1.587-5.920)	0.001
TNM stage (III + IV vs. I + II)	3.72 (1.933-7.177)	<0.001
MULTIVARIATE ANALYSIS		
3-miRNA signature (high-risk vs. low-risk)	2.13 (1.108-4.107)	0.023
TNM stage (III + IV vs. I + II)	3.37 (1.733-6.651)	<0.001

## Discussion

Using a custom microarray containing 1,094 probes for human miRNAs, we detected microRNA profiles in 63 ICC patients, which is the largest sample size in such studies of ICC so far. The relationships between microRNA expression levels and survival, as well as other clinical features in these patients, were analyzed. Our data showed that 158 miRNAs (77 up-regulated miRNAs and 81 down-regulated) were differentially expressed in tumor tissues compared with NIBD, and a 30-miRNA signature was established for discriminating ICC from NIBD with 100% accuracy. More important, we established a 3-miRNA signature that was an independent predictor for the survival of patients with ICC.

Comparing the 3-mRNA signature with the 30-miRNA signature, we found that miR-675-5p and miR-338-3p were shared between the two signatures, while miR-652-3p was not included in the 30-miRNA signature. The reason for this phenomenon was that miRNAs with more than 2-fold change were selected for establishing the signature for distinguishing ICC from NIBD, while those with more than 1.5-fold change were chosen for constructing the prognostic signature. Consequently, miR-652-3p (1.52-fold change) was not presented in the 30-miRNA signature.

In the literature, there are only a few studies on miRNA profiles in human intrahepatic cholangiocarcinoma. In 2009, Chen et al. [18] identified a 38-miRNA signature by PCR array (containing only 156 miRNAs), which could discriminate ICC from normal cholangiocyte. When compared with the 38-miRNA signature, we found that our 30-miRNA signature only shared three miRNAs in common with the 38-miRNA signature. Specifically, miR-338 and let-7a were consistently reduced in ICC in the two studies, while the miR-103 was up-regulated in Chen’s study and downregulated in our study. The miRNA discrepancy between the two signatures might be caused by the different number of miRNAs (1,094 versus 156) and cases (63 versus 27), the different clinical features of patients, or the particular samples used in the two different studies. In the same year, Selaru et al. [28] performed a miRNA microarray (containing probes for 470 unique human miRNAs) on five pairs of primary cholangiocarcinoma (CCA) and normal bile duct specimens (NBDs), and listed the top 10 up-regulated and 10 down-regulated miRNAs. Not surprisingly, only one miRNA (hsa-miR-513) was consistent with our differentially expressed miRNAs, suggesting that the biological characteristic of CCA is different from ICC. In 2012, another study reported that a 23-miRNA signature, identified in two subtypes of 23 ICCs, was associated with the survival of patients with hepatocellular carcinoma [19], demonstrating the shared properties between ICC and HCC. In October of this year, Plieskatt et al. reported a miRNA profiling study on 15 ICCs caused by liver fluke *Opisthorchis viverrini*, in which they focused on the relationship between miRNA expression profiles and histological grades, as well as subtypes of ICC, and compared the differential miRNA expression profiles between ICC and liver tissues [20]. Despite the study’s similar investigation into miRNAs in ICC, there was no comparability between our study and Plieskatt’s study.

However, there have been no reports on a miRNA signature associated with survival of ICC patients to date. As the survival-related miRNA signature may potentially help clinicians identify patients at a higher risk or lower risk and choose the appropriate treatment, it will be very important in improving the prognosis and treatment of patients with ICC. With the data from our custom microarray, we have developed the first survival-related miRNA signature, consisting of three miRNAs, which is significantly associated with OS and DFS in 63 patients with ICC. The novel 3-miRNA signature was statistically significant in both univariate and multivariate Cox regression analyses (Table 3), which suggest that the 3-miRNA signature may be a useful prognostic indicator for survival in patients with ICC and may have clinical utility. Of the three miRNAs, miR-675 has been reported to be over expressed and correlates with survival of pancreatic cancer patients [29], and miR-338-3p was down-regulated and associated with prognosis of colorectal carcinoma [30], which supports a similar expression pattern for these miRNAs in other cancers. However, miR-338 has been reported to be over-expressed and linked to a poor outcome in gastric cancer [31], which is inconsistent with the pattern we observed for miR-338 in ICC. This discrepancy might be caused by the different kinds of cancers in the two studies (ICC vs. gastric cancer). Considering that the three miRNAs (miR-675-5p, miR-652-3p and miR-338-3p) are highly dysregulated in ICC and other cancers, these miRNAs may play an important role in ICC carcinogenesis. Therefore, we are conducting further studies on the biological function and the regulation of miR-675-5p and miR-652-3p expression in ICC cells.

With univariate Cox regression analysis, AFP concentration was found to be associated with overall survival. Kaplan-Meier curve analysis also showed that patients with high-level AFP had much poorer survival than those with low-level AFP (*P* <0.05, data not shown). However, no report has shown that high-level AFP is correlated with overall survival of ICC patients. Moreover, we found that all of the four patients with higher AFP concentration died within 18 months after surgery. Therefore, we speculate that this significant correlation might be caused by the statistical bias because of the very limited sample size of patient with high-level AFP.

## Conclusions

In conclusion, we have established a 30-miRNA signature that can discriminate ICC tissues from normal intrahepatic bile duct and a 3-miRNA signature that is associated with the survival of ICC patients after resection. Importantly, the 3-miRNA signature may be a potential new biomarker for the prognosis of patients with ICC. In addition, the miRNAs identified in the two signatures might be involved in ICC carcinogenesis. Our results warrant further studies on these miRNAs that will shed light on the mechanisms associated with ICC pathogenesis and progression.

## Abbreviations

AFP: Alpha-fetoprotein
ALT: Alanine aminotransferase
AST: Aspartate aminotransferase
CA19-9: Carbohydrate antigen 19–9
CCA: Cholangiocarcinoma
CEA: Carcinoembryonic antigen
CI: Confidence interval
DFS: Disease-free survival
EMT: Epithelial to mesenchymal transition
FDR: False discovery rate
HBsAg: Hepatitis B surface antigen
HCC: Hepatocellular carcinoma
HCL: Hierarchical clustering analysis
HR: Hazard ration
ICC: Intrahepatic cholangiocarcinoma
Int.: Intensity
MEV: Multi experiment viewer
miRNAs: microRNAs
MRI: Magnetic resonance imaging
NBDs: Normal bile duct specimens
NIBD: Normal intrahepatic bile duct
OS: Overall survival
qRT-PCR: Quantitative real-time RT-PCR
RT: Reverse transcription
SAM: Significance analysis of microarrays
TBIL: Total bilirubin
